# Supplementary material for: MBTPS2 mutations cause defective regulated intramembrane proteolysis in X-linked osteogenesis imperfecta
Source: Nat Commun. 2016 Jul 6;7:11920. doi: 10.1038/ncomms11920 (PMC4935805; doi:10.1038/ncomms11920)
Supplement: Supplementary Information — Supplementary Figures 1-9, Supplementary Tables 1-8 and Supplementary References [file ncomms11920-s1.pdf]

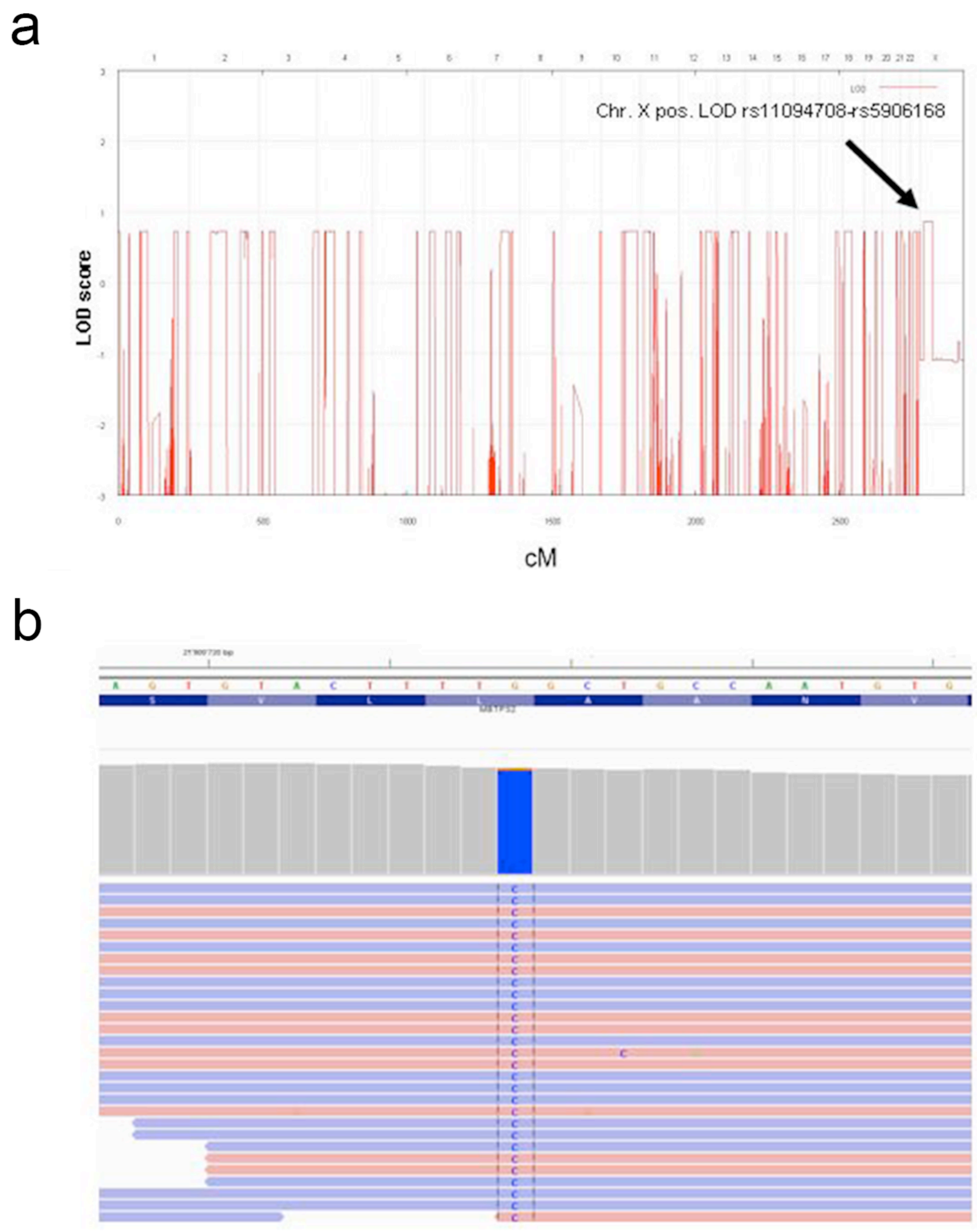


**Supplementary Figure 1** Linkage analysis of Family II. (**a**) Parametric multipoint LOD score in family II is shown. Genetic distances in cM and the LOD scores are shown on the x-axis and on the y-axis, respectively. On the X chromosome the 30 Mb region of positive LOD score between rs11094708 and rs5906168 encompasses *MBTPS2* (**Supplementary Table 5**). (**b**) Sequence read alignments for *MBTPS2* obtained with MiSeqReporter Software (Illumina).

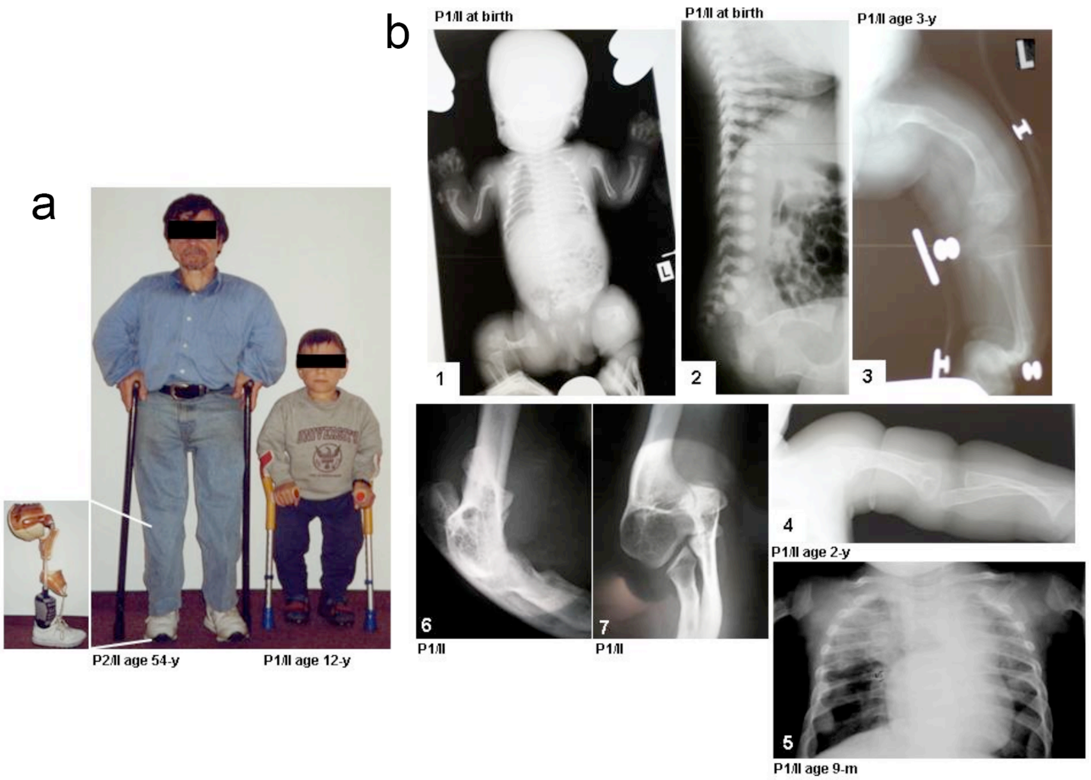


**Supplementary Figure 2** Clinical and radiological features of Family II. (**a**) Clinical features of affected individuals P1/II and P2/II from family II showing consistent rhizomelia of upper and lower extremities. (**b**) Radiological features of affected Proband 1/II from Family II. (1) Newborn full-body radiography depicting poor skull ossification, small bell shaped thorax, slender ribs; long bones are thin, bent and osteopenic with areas of both thin and thick cortices; prenatal fracture of left femur. (2) Thoracic and lumbar spine radiographs show scoliosis with abnormal ribs, ossification and thin cortices. Vertebral bodies are flat and biconcave, with significant osteopenia. (3) Bones of lower limb are markedly deformed and consolidated fractures (3) are present; (4) Arm/shoulder radiography at age 2 years showing thin, bent and osteopenic long bones; (5) Discontinuously beaded ribs; (6) and (7) Lateral and frontal view, respectively, of the elbow showing typical “popcorn” epiphysis.

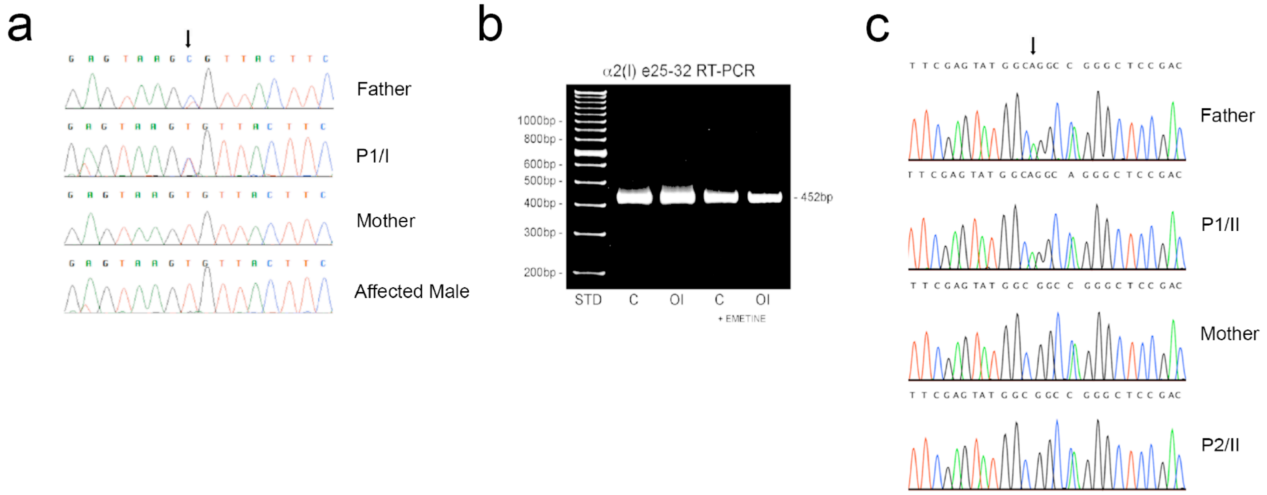


**Supplementary Figure 3** Exclusion of functional mutations in other OI genes. (**a**) Analysis of genes in which mutations have been associated with osteogenesis imperfecta (OI) revealed a heterozygous *COL1A2* IVS30+6T>C transition in Family **I**, which does not alter the exonic splice donor consensus sequence. Both the father and OI proband were heterozygous for the substitution, while the proband's obligate carrier mother and all the other five affected male family members had only normal sequence. (**b**) Only normal splice products were identified in normal control (C) and P1/I (OI) samples by RT-PCR analysis. No alternative splice products were identified in cells treated with emetine (+) to inhibit nonsense-mediated decay. (**c**) Sequence analysis of Family **II** demonstrated a heterozygous *COL1A1* c.4018G>A transition, predicting a p.G1340S substitution in the C-propeptide of type I collagen α1(**I**) chains, in the proband P1/**II** and his healthy father. This SNP has an allele frequency of 0.1% in dbSNP, 0.07% in ESP and 0.3% in ExAC, and is absent in the mother and affected uncle (P2/**II**) of proband P1/**II**. Furthermore, there is a glycine at this position in 17 of 25 species analyzed, while in the other 8 species a serine is present.

a

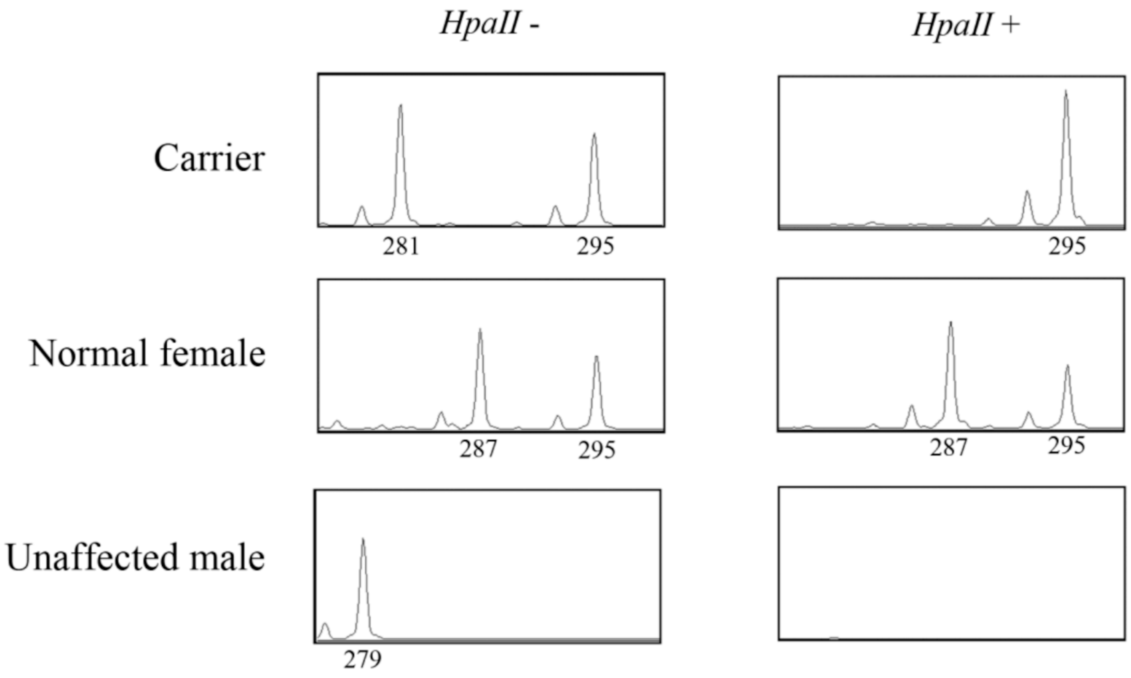


b

| Individual | Gender | Status | Xi-pattern |
|---|---|---|---|
| III1 | Female | Obligate carrier | 100:0 |
| III5 | Female | Obligate carrier | 100:0 |
| III7 | Female | Normal female | 41:59 |
| IV5 | Female | Normal female | 63:37 |
| IV13 | Female | Normal female | 84:16 |
| IV18 | Female | Obligate carrier | 100:0 |
| IV21 | Female | Obligate carrier | 100:0 |
| IV23 | Female | Obligate carrier | 100:0 |
| IV19 | Male | Unaffected male | - |

**Supplementary Figure 4** X-inactivation studies of Family I. (**a**) X-inactivation analysis: *Hpa*II - indicates no prior *Hpa*II digestion, and *Hpa*II + indicates prior *Hpa*II digestion. (**b**) Levels of X-inactivation of female members are shown. Status of carriers is indicated post-mutation determination.

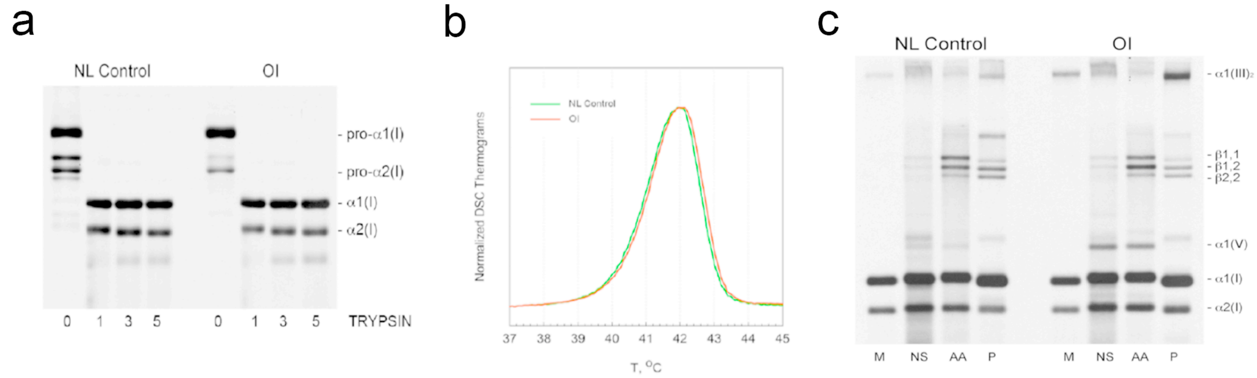


**Supplementary Figure 5** Collagen Biochemical analysis for Proband 1/I. (**a**) No difference in collagen triple helix structural integrity is observed between normal (NL) control fibroblast and proband (OI) secreted procollagens, as determined by a collagen protease susceptibility assay. (**b**) Differential scanning calorimetry (DSC) shows normal collagen thermal stability, consistent with the absence of overmodification or structural abnormalities in proband collagen. (**c**) Type I collagen deposition into patient fibroblast-derived extracellular matrix in culture. Following a 24 hr pulse of labeled collagen by post-confluent cultures, incorporated collagens were serially extracted from the media (M), neutral salt (NS), acid soluble (AA, immaturely crosslinked) and pepsin soluble (P, maturely crosslinked) fractions of the matrix[1]. Decreased type I collagen deposition by proband fibroblasts, compared to normal control fibroblasts, is visually demonstrated by a relative increase in the amount of type III collagen extracted from fibroblast-derived extracellular matrix. The amount of collagen in each fraction was measured by densitometry and normalized to sample volume.

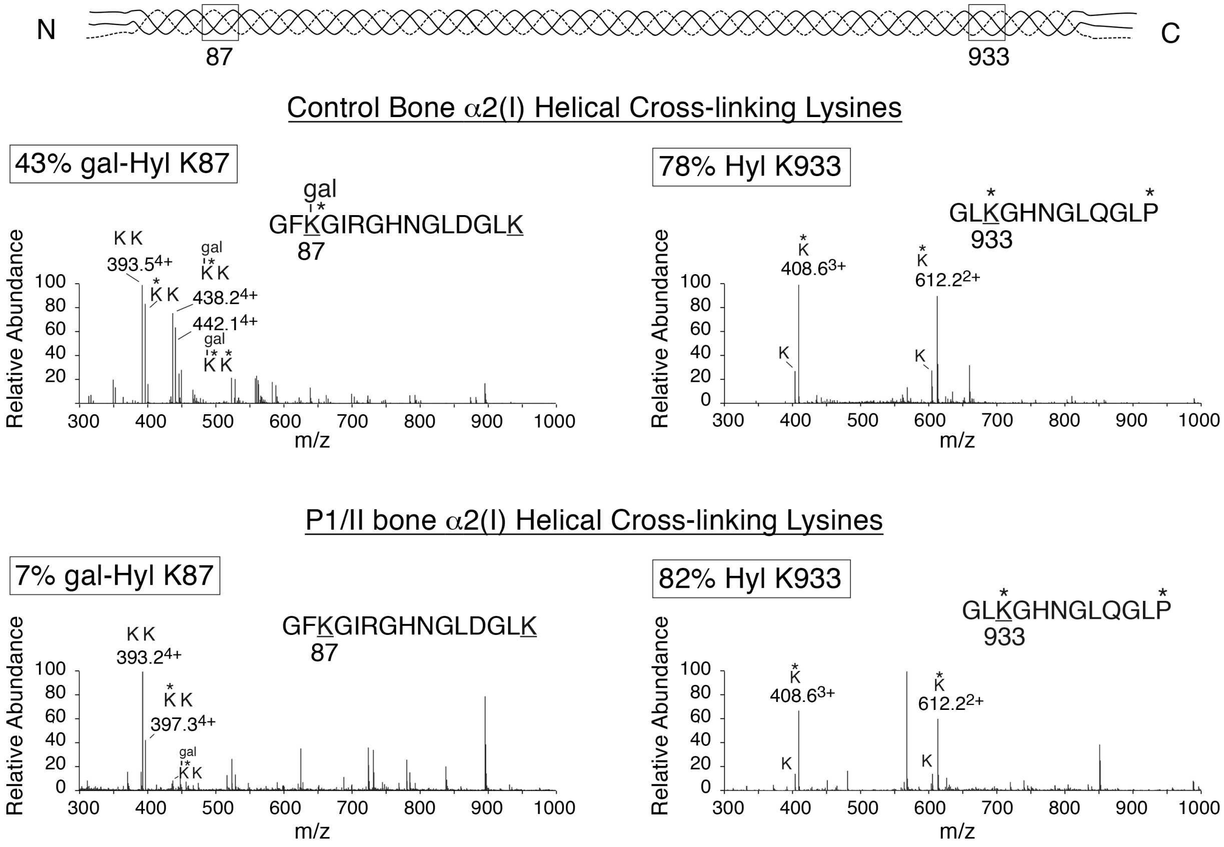


**Supplementary Figure 6** P1/II Bone tissue α2(I) collagen mass spectrometry. Mass spectrometric analysis of bacterial collagenase-digested peptides of control and P1/II bone type I collagen. Hydroxylation and consequent glycosylation of α2(I)K87 residues, involved in crosslink formation, are decreased in the proband sample by more than one-half. Hydroxylation of α2(I)K933 residues is normal, compared to normal control bone. K, lysine; K*, hydroxylysine; gal, galactosyl.

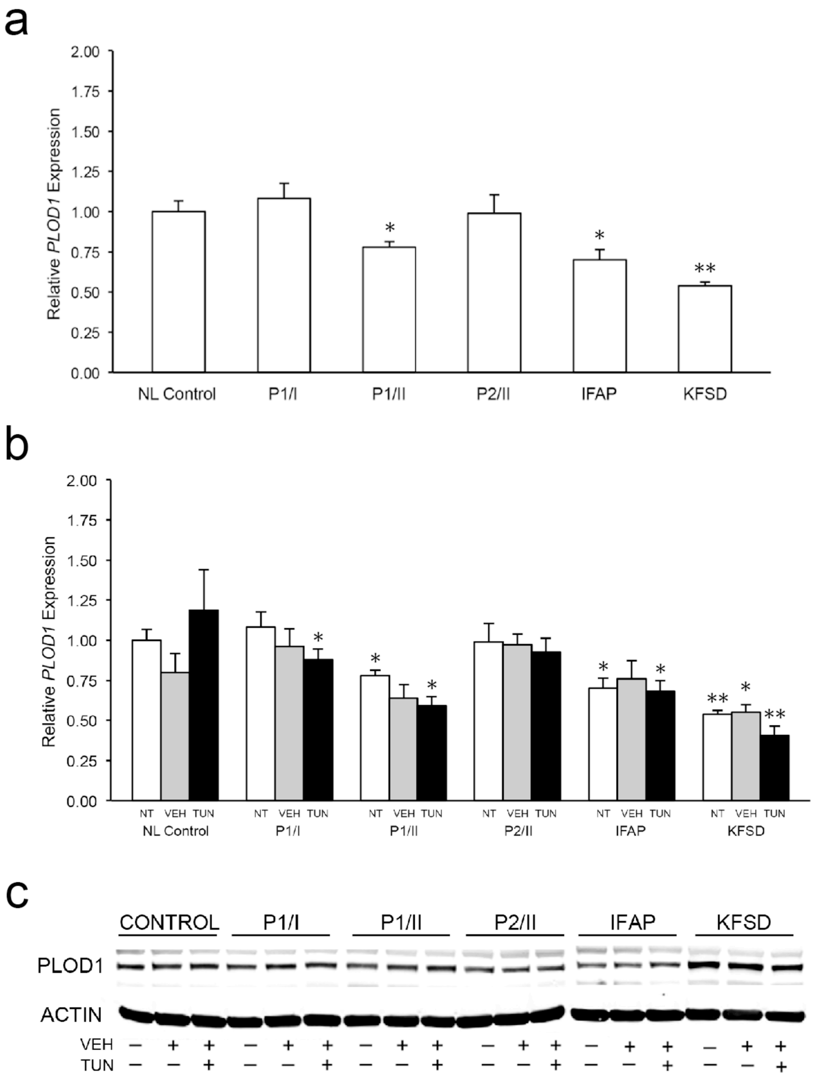


**Supplementary Figure 7** Expression of *PLOD1*/LH1 in proband cell lines. (**a**) Quantitation of *PLOD1* transcripts in OI proband (P1/I, P1/II and P2/II), IFAP and KFSD fibroblasts relative to normal (NL) control fibroblasts. (**b**) Expression of *PLOD1* transcripts in the absence (NT, no treatment) or presence of ER stress inducer tunicamycin (TUN), or vehicle only (VEH). (**c**) Immunoblots of PLOD1/LH1 protein in OI proband (P1/I, P1/II and P2/II), IFAP, KFSD and normal control fibroblasts in the absence (NT, no treatment) or presence of ER stress inducer tunicamycin (TUN), or vehicle only (VEH). *P < 0.05, **P< 0.001 compared to control with equivalent treatment conditions by t-test; error bars, s.d.

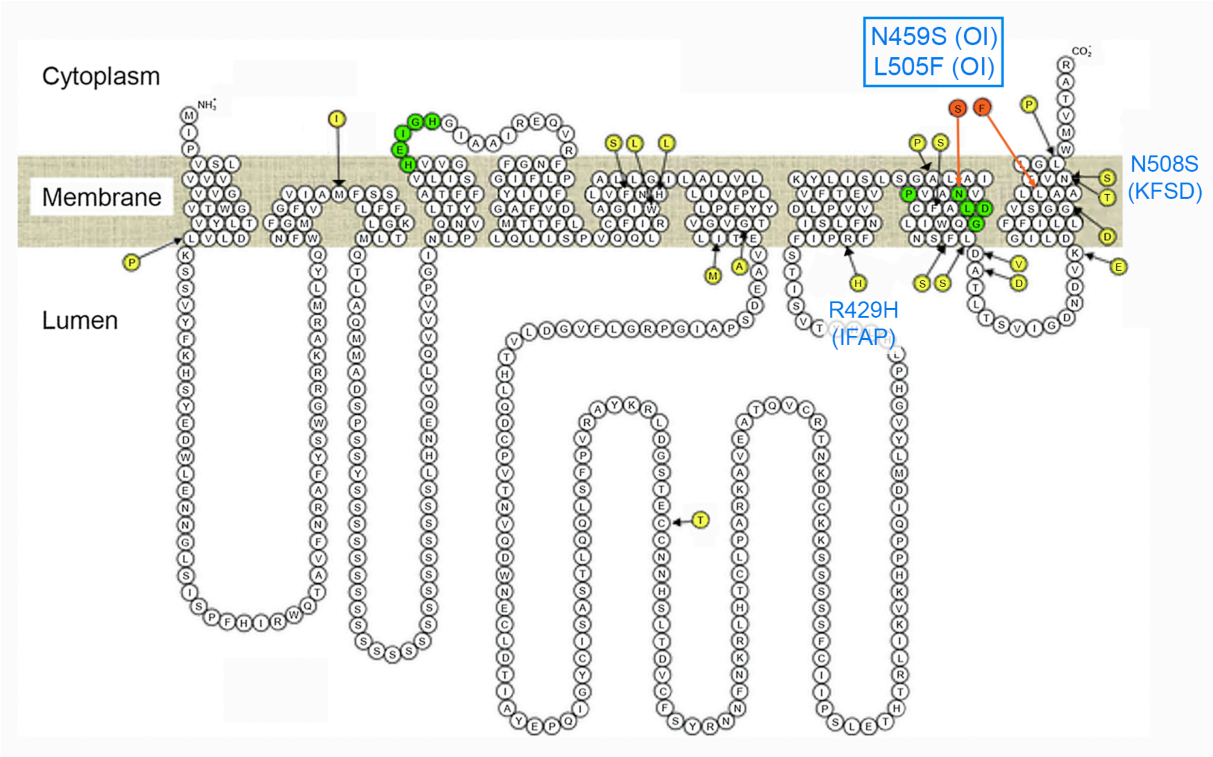


**Supplementary Figure 8** Map of *MBTPS2* mutations. Map of reported *MBTPS2* missense mutations (LOVD gene homepage for *MBTPS2* at https://grenada.lumc.nl/LOVD2/MR/home.php?select_db=MBTPS2)[2-6] resulting in amino acid residue substitutions. Green residues represent the HEIGH and LDG/NPDG motifs, which are required for proteolytic activity via coordination of the active site metal atom. Yellow residues represent MBTPS2 substitutions in probands with IFAP, BRESHECK, KFSD and Olmsted syndromes. Red residues, which are adjacent or contained within the active site, are substitutions found in X-linked osteogenesis imperfecta probands reported in this study. Amino acid substitutions labeled in blue represent cell lines used for functional studies in this report. Substitution map was modified from Bornholdt, *et al*[7].

Fig. 3b

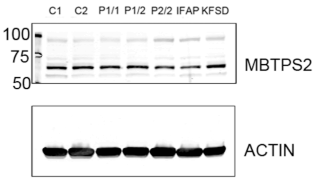


Fig. 4a

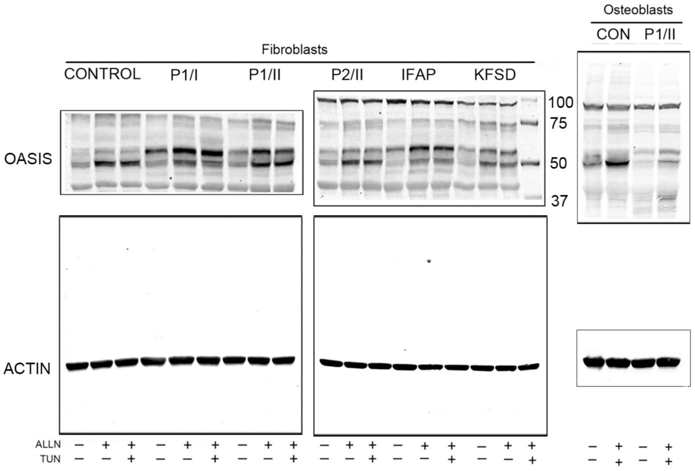


Fig. 5c

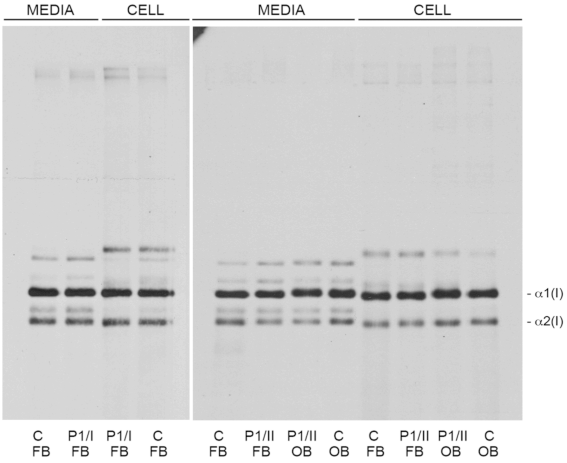


Fig. 7b

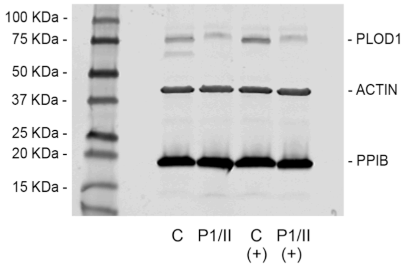


**Supplementary Figure 9** Full-length immunoblots and autoradiographs presented in the main figures.

**Supplementary Table 1** Clinical features of affected individuals with X-linked osteogenesis imperfecta.

| | IV7 | IV9 | IV17 | IV27 | P1/I (V7) | V13 | P1/II | P2/II |
|---|---|---|---|---|---|---|---|---|
| **OI type** | III | III | III | III | III | III | III/IV | III/IV |
| **Age (yr)** | 39 | 36 | 36 | 30 | 2 | 15 | 26 | 68 |
| **Gestational age** | Term | Term | Term | Term | Term | Term | Term | Term |
| **Delivery type** | NL | NL | NL | NL | C/S | C/S | NL | NL |
| **Birth weight** | Normal | Normal | Normal | Normal | 2,860 g | 3,600 g | 2970 g | Normal |
| **Birth Length** | - | - | - | - | - | - | 46 cm | - |
| **Prenatal fractures** | Yes | Yes | Yes | Yes | Yes | Yes | Yes | - |
| **Weight (kg)** | 23 | 70 | 36 | 70 | 10.0 | 29 | 37 | 45 |
| **Length (cm)** | 81.5 | 134 | 135.5 | 140 | 76.5 | 110 | 110 | 105 |
| **Head circumference (cm)** | 55 | 57.5 | 54.5 | 58 | 47.8 | 52.5 | - | - |
| **Color of sclera** | White | White | White | White | Blue | White | White | White |
| **Dentinogenesis imperfecta** | No | No | No | No | No | No | No | No |
| **Joint hypermobility** | No | No | No | No | No | No | No | No |
| **Hearing impairment** | No | No | No | No | No | No | No | No |
| **Pectus deformities** | Pectus carinatum | No | Pectus carinatum | No | Pectus carinatum | No | No | Pectus excavatum |
| **Bowing of upper extremities** | Yes | Yes | Yes | Yes | No | Yes | Yes | Yes |
| **Bowing of lower extremities** | Yes | Yes | Yes | Yes | Yes | Yes | Yes | Yes |
| **Scoliosis** | Yes | No | Yes | No | No | No | Yes | Yes |
| **Motility** | Unable to bear own weight | Unable to walk but can drive motorcycle | Unable to bear own weight | Ambulate by using cane | Walk with supports | Unable to bear own weight | Walk with support 5-10 m Wheel-chair bound | Slow and difficult ambulation with crutches |
| **Intelligence** | Normal | Normal | Normal | Normal | Normal | Normal | Normal | Normal |

C/S Caesarean Section, NL normal labor, - not available

**Supplementary Table 2** Urinary pyridinoline crosslink profile.

| | P1/I | IV17/I | IV18/I (carrier) | P1/II | P2/II | II3/II (carrier) | R429H (IFAP) | R429H (carrier) |
|---|---|---|---|---|---|---|---|---|
| LP/HP ratio[#] | 0.319[§] | 0.45[*] | 0.17[†] | 0.45[*] | 0.305[*] | 0.176[†] | 0.198[§] | 0.19[†] |

[#] Single measurements are reported.
[§] The normal control value for healthy children (age 5-8 years) is 0.208 ± 0.03, as reported by Kraenzlin *et al.*[8]
[*] The normal control value for healthy adult males (age 20-60 years) is 0.211 ± 0.008.[8]
[†] The normal control value for healthy adult females (age 20-49 years) is 0.206 ± 0.008.[8]

**Supplementary Table 3** LOD score of linkage analysis for Family I.

| Locus | Physical distance (Primary Assembly, Mb) | Genetic distance (Marshfield, cM) | LOD score at the recombination fraction | | | | | | | |
|---|---|---|---|---|---|---|---|---|---|---|
| | | | 0.000 | 0.010 | 0.050 | 0.100 | 0.200 | 0.300 | 0.400 | 0.500 |
| DXS1060 | 5.4 M | 15.12 | -∞ | -1.680 | -0.390 | 0.071 | 0.367 | 0.374 | 0.234 | 0.000 |
| DXS8051 | 9.5 M | 17.29 | -∞ | -1.087 | 0.167 | 0.581 | 0.774 | 0.665 | 0.391 | 0.000 |
| DXS7108 | 10.2 M | 18.37 | -∞ | 0.910 | 1.450 | 1.540 | 1.380 | 1.030 | 0.560 | 0.000 |
| DXS1224 | 13.2 M | 21.23 | 0.95, (3.31) | 0.92, (3.26) | 0.85, (3.04) | 0.86, (2.76) | 0.85, (2.16) | 0.69, (1.49) | 0.40, (0.76) | 0.000 |
| DXS8022 | 13.9 M | 22.18 | -1.500 | -1.320 | -0.930 | -0.670 | -0.380 | -0.200 | -0.090 | 0.000 |
| DXS7943 | 14.1 M | NA | NA | NA | NA | NA | NA | NA | NA | NA |
| DXS987 | 14.7 M | 22.18 | 0.95, (3.31) | 0.92, (3.26) | 0.85, (3.04) | 0.86, (2.76) | 0.85, (2.16) | 0.69, (1.49) | 0.40, (0.76) | 0.000 |
| DXS9902 | 15.3 M | 22.18 | 0.650 | 0.920 | 1.250 | 1.320 | 1.200 | 0.9) | 0.490 | 0.000 |
| DXS1053 | 15.5 M | 22.18 | -1.500 | -1.320 | -0.930 | -0.670 | -0.380 | -0.200 | -0.090 | 0.000 |
| DXS7985 | 16.5 M | NA | 0.000 | 0.000 | 0.000 | 0.000 | 0.000 | 0.000 | 0.000 | 0.000 |
| DXS8036 | NA | 22.72 | 0.000 | 0.000 | 0.000 | 0.000 | 0.000 | 0.000 | 0.000 | 0.000 |
| DXS8019 | 17.7 M | 23.26 | 0.350 | 0.620 | 0.970 | 1.070 | 0.990 | 0.750 | 0.410 | 0.000 |
| DXS999 | 18.83 M | 23.26 | 0.95, (3.31) | 0.92, (3.26) | 0.85, (3.04) | 0.86, (2.76) | 0.85, (2.16) | 0.69, (1.49) | 0.40, (0.76) | 0.000 |
| DXS1229 | 20.5 M | 27.59 | 0.033 | 0.032 | 0.027 | 0.022 | 0.013 | 0.007 | 0.002 | 0.000 |
| DXS7101 | 21.7 M | 25.97 | 0.95, (3.31) | 0.92, (3.26) | 0.85, (3.04) | 0.86, (2.76) | 0.85, (2.16) | 0.69, (1.49) | 0.40, (0.76) | 0.000 |
| DXS7105 | 22.34 M | 25.97 | 0.653 | 0.917 | 1.249 | 1.324 | 1.194 | 0.899 | 0.489 | 0.000 |
| DXS7593 | 22.4 M | 25.97 | 0.650 | 0.920 | 1.250 | 1.320 | 1.190 | 0.900 | 0.490 | 0.000 |
| DXS1226 | 25.5 M | 27.59 | -1.350 | -1.180 | -0.820 | -0.600 | -0.350 | -0.200 | -0.090 | 0.000 |
| DXS7110 | 23.11 M | 29.22 | 0.770 | 0.733 | 0.658 | 0.658 | 0.632 | 0.464 | 0.187 | 0.000 |
| DXS989 | 23.18 M | 29.76 | 0.804 | 0.766 | 0.686 | 0.679 | 0.643 | 0.467 | 0.186 | 0.000 |

| Locus | Physical distance (Primary Assembly, Mb) | Genetic distance (Marshfield, cM) | LOD score at the recombination fraction | | | | | | | |
|---|---|---|---|---|---|---|---|---|---|---|
| | | | 0.000 | 0.010 | 0.050 | 0.100 | 0.200 | 0.300 | 0.400 | 0.500 |
| DXS1202 | 26.47 M | 30.3 | 0.487 | 0.748 | 1.067 | 1.128 | 0.976 | 0.667 | 0.277 | 0.000 |
| DXS7106 | 27.8 M | 30.84 | -1.370 | -1.202 | -0.842 | -0.614 | -0.360 | -0.204 | -0.090 | 0.000 |
| DXS9896 | 29.3 M | 30.84 | -1.050 | -0.883 | -0.545 | -0.343 | -0.146 | -0.053 | -0.010 | 0.000 |
| DXS1214 | 31.2 M | 33.54 | -1.040 | -0.881 | -0.543 | -0.342 | -0.146 | -0.053 | -0.010 | 0.000 |
| DXS1067 | 31.8 M | 33.54 | -∞ | -1.263 | -0.620 | -0.297 | 0.032 | 0.107 | 0.052 | 0.000 |
| DXS8090 | 36.95 M | 36.79 | -∞ | -1.384 | -0.110 | 0.328 | 0.574 | 0.525 | 0.318 | 0.000 |
| DXS1069 | 38.62 M | 37.33 | -∞ | 0.399 | 0.949 | 1.056 | 0.939 | 0.648 | 0.270 | 0.000 |
| DXS1068 | 38.9 M | 37.33 | -∞ | 0.445 | 0.988 | 1.087 | 0.956 | 0.656 | 0.272 | 0.000 |
| DXS993 | 41.1 M | 42.21 | -∞ | -1.085 | 0.170 | 0.585 | 0.779 | 0.668 | 0.389 | 0.000 |
| DXS991 | 55.5 M | 52.5 | -∞ | -3.373 | -1.383 | -0.621 | -0.024 | 0.159 | 0.142 | 0.000 |
| DXS986 | 79.3 M | 57.37 | -∞ | -5.076 | -2.387 | -1.324 | -0.425 | -0.066 | 0.043 | 0.000 |
| DXS990 | 93 M | 60.62 | -∞ | -2.878 | -1.823 | -1.298 | -0.748 | -0.421 | -0.186 | 0.000 |
| DXS1106 | 102.7 M | 66.58 | -1.040 | -0.881 | -0.543 | -0.342 | -0.146 | -0.053 | -0.010 | 0.000 |
| DXS8055 | 114.6 M | 70.91 | 0.033 | 0.032 | 0.027 | 0.022 | 0.013 | 0.007 | 0.002 | 0.000 |
| DXS1001 | 119.8 M | 75.79 | -∞ | -3.085 | -1.113 | -0.374 | 0.173 | 0.303 | 0.227 | 0.000 |
| DXS1047 | 129 M | 82.07 | -∞ | -1.382 | -0.127 | 0.287 | 0.487 | 0.407 | 0.218 | 0.000 |
| DXS1227 | 140.8 M | 88.33 | -∞ | -1.271 | -0.026 | 0.377 | 0.554 | 0.444 | 0.217 | 0.000 |
| DXS8043 | 144 M | 94.22 | -∞ | -0.276 | 0.334 | 0.518 | 0.567 | 0.462 | 0.268 | 0.000 |
| DXS8091 | 147.6 M | 96.14 | 0.334 | 0.328 | 0.306 | 0.277 | 0.217 | 0.153 | 0.081 | 0.000 |
| DXS1073 | 153.8 M | 102.35 | -∞ | -5.265 | -2.585 | -1.528 | -0.627 | -0.234 | -0.055 | 0.000 |

**Supplementary Table 4** NGS filtering criteria for Family I.

| Filtering criteria | Number of variants after each filtering | |
|---|---|---|
| | substitutions | indels |
| Total | 7,527 | 4,369 |
| Nonsynonymous | 25 | 0 |
| Not in dbSNP | 2 | 0 |
| Not in Thai controls | 1 | 0 |

**Supplementary Table 5** Linkage analysis LOD scores for Family II.

| SNP rs number | Physical Map Position (in KB) | LOD | alpha | HLOD |
|---|---|---|---|---|
| rs11094708 | 13.875 | -1.0877 | 1 | 0 |
| rs5980314 | 13.892 | 0.3581 | 0 | 0.3581 |
| rs5980326 | 13.919 | 0.7768 | 0 | 0.7768 |
| rs5950870 | 14.431 | 0.867 | 0 | 0.867 |
| rs17320692 | 15.207 | 0.867 | 0 | 0.867 |
| rs6629282 | 15.814 | 0.867 | 0 | 0.867 |
| rs12395191 | 16.312 | 0.867 | 0 | 0.867 |
| rs936424 | 16.861 | 0.867 | 0 | 0.867 |
| rs5909364 | 17.429 | 0.867 | 0 | 0.867 |
| rs16981405 | 17.686 | 0.867 | 0 | 0.867 |
| rs5951504 | 18.551 | 0.867 | 0 | 0.867 |
| rs6528055 (MBTPS2) | 19.053 | 0.867 | 0 | 0.867 |
| rs5951534 | 19.501 | 0.867 | 0 | 0.867 |
| rs972377 | 19.995 | 0.867 | 0 | 0.867 |
| rs5970712 | 20.511 | 0.867 | 0 | 0.867 |
| rs7886064 | 21.054 | 0.867 | 0 | 0.867 |
| rs11094971 | 21.738 | 0.867 | 0 | 0.867 |
| rs5944695 | 22.122 | 0.867 | 0 | 0.867 |
| rs4132881 | 22.652 | 0.867 | 0 | 0.867 |
| rs4898240 | 23.096 | 0.867 | 0 | 0.867 |
| rs4344230 | 23.515 | 0.867 | 0 | 0.867 |
| rs5986678 | 24.038 | 0.867 | 0 | 0.867 |
| rs5926405 | 24.542 | 0.867 | 0 | 0.867 |
| rs12013038 | 25.092 | 0.867 | 0 | 0.867 |
| rs6630659 | 25.595 | 0.867 | 0 | 0.867 |
| rs1813566 | 26.095 | 0.867 | 0 | 0.867 |
| rs5972125 | 26.662 | 0.867 | 0 | 0.867 |
| rs6628477 | 27.021 | 0.867 | 0 | 0.867 |
| rs1034948 | 27.528 | 0.867 | 0 | 0.867 |
| rs5927624 | 28.026 | 0.867 | 0 | 0.867 |
| rs4642801 | 28.529 | 0.867 | 0 | 0.867 |
| rs1471174 | 29.028 | 0.867 | 0 | 0.867 |
| rs5971622 | 29.534 | 0.867 | 0 | 0.867 |
| rs5972677 | 30.031 | 0.867 | 0 | 0.867 |
| rs12559939 | 30.509 | 0.867 | 0 | 0.867 |
| rs2455623 | 31.125 | 0.867 | 0 | 0.867 |
| rs4402033 | 31.587 | 0.867 | 0 | 0.867 |
| rs5973287 | 32.105 | 0.867 | 0 | 0.867 |
| rs5927399 | 32.63 | 0.867 | 0 | 0.867 |

| | | | | |
|---|---|---|---|---|
| rs5973518 | 33.086 | 0.867 | 0 | 0.867 |
| **SNP rs number** | **Physical Map Position (in KB)** | **LOD** | **alpha** | **HLOD** |
| rs5973651 | 33.583 | 0.8669 | 0 | 0.8669 |
| rs12006776 | 33.887 | 0.8669 | 0 | 0.8669 |
| rs5917471 | 34.823 | 0.867 | 0 | 0.867 |
| rs5918462 | 35.081 | 0.867 | 0 | 0.867 |
| rs5917599 | 35.522 | 0.867 | 0 | 0.867 |
| rs964528 | 36.022 | 0.867 | 0 | 0.867 |
| rs1982836 | 36.528 | 0.867 | 0 | 0.867 |
| rs17144380 | 37.056 | 0.867 | 0 | 0.867 |
| rs5963178 | 37.521 | 0.867 | 0 | 0.867 |
| rs1243792 | 38.042 | 0.867 | 0 | 0.867 |
| rs2859010 | 38.523 | 0.867 | 0 | 0.867 |
| rs12843591 | 39.113 | 0.867 | 0 | 0.867 |
| rs12557513 | 39.538 | 0.867 | 0 | 0.867 |
| rs17214797 | 40.059 | 0.867 | 0 | 0.867 |
| rs2153338 | 40.524 | 0.867 | 0 | 0.867 |
| rs3859956 | 41.024 | 0.867 | 0 | 0.867 |
| rs1040415 | 41.51 | 0.867 | 0 | 0.867 |
| rs5905916 | 42.05 | 0.867 | 0 | 0.867 |
| rs13440967 | 42.527 | 0.867 | 0 | 0.867 |
| rs570515 | 43.038 | 0.867 | 0 | 0.867 |
| rs5906168 | 43.307 | -1.0877 | 1 | 0 |

**Supplementary Table 5** Parametric multipoint linkage analysis in Family II under the assumption of a recessive mode of inheritance. The region of positive LOD score on the X chromosome is shown. SNP markers are given with the official rs number. The region of positive LOD score between SNP rs11094708 and rs5906168 encompasses *MBTPS2* (rs6528055) and is defined by 1545 SNPs, in total. Due to space concern, physical map positions for 61 SNPs spaced approximately every 500 Kb are shown. The entire data set for 1545 SNPs is available upon request. Each row indicates the estimated multipoint LOD score at a particular location of the physical map for a given SNP. Alpha indicates the estimate proportion of linked families (since only one family has been investigated, the proportion is either 0.000 or 1.000), and HLOD indicates the corresponding maximum heterogeneity LOD score.

**Supplementary Table 6** Prediction of Mutation Deleterious Effects

| | | **Mutation** | |
|---|---|---|---|
| | | **c.1376A>G** | **c.1515G>C** |
| | | **N459S** | **L505F** |
| **Prediction Programs** | PolyPhen2 | Probably damaging | Probably damaging |
| | SIFT | Damaging | Damaging |
| | Provean | Deleterious | Deleterious |
| | MutationTaster | Disease causing | Disease causing |
| **Database of Genetic Variants** | LOVD | None | None |
| | ExAC | None | None |
| | EVS | None | None |
| | 1000G | None | None |
| | dbSNP build 144 | None | None |

**Supplementary Table 7** ATF6 Luciferase Reporter Assays

| Plasmid | Experiment | No tunicamycin | | | | | Tunicamycin | | | |
|---|---|---|---|---|---|---|---|---|---|---|
| | | Luciferase | Average of wild-type luciferase | Normalized by A-WT | Average | Corrected SD | Luciferase | Normalized by A-WT | Average | Corrected SD |
| Vector | 1 | 0.0558 | | 0.3075 | 0.2821 | 0.0634 | 0.1753 | 0.9660 | 1.0667 | 0.2310 |
| | 1 | 0.0460 | | 0.2535 | | | 0.1887 | 1.0399 | | |
| | 1 | 0.0628 | | 0.3461 | | | 0.2143 | 1.1809 | | |
| | 2 | 0.0372 | | 0.1901 | | | 0.2485 | 1.2698 | | |
| | 2 | 0.0479 | | 0.2448 | | | 0.2495 | 1.2749 | | |
| | 2 | 0.0686 | | 0.3505 | | | 0.1309 | 0.6689 | | |
| Wild type | 1 | 0.1682 | | 0.9269 | 1.0000 | 0.1048 | 2.2187 | 12.2265 | 15.9875 | 3.6540 |
| | 1 | 0.1875 | | 1.0332 | | | 2.5717 | 14.1717 | | |
| | 1 | 0.1887 | 0.1815 | 1.0399 | | | 2.2796 | 12.5621 | | |
| | 2 | 0.2290 | | 1.1702 | | | 4.1945 | 21.4333 | | |
| | 2 | 0.1872 | | 0.9566 | | | 3.3016 | 16.8707 | | |
| | 2 | 0.1709 | 0.1957 | 0.8733 | | | 3.6519 | 18.6607 | | |
| N459S | 1 | 0.0773 | | 0.4260 | 0.5859 | 0.1425 | 0.4409 | 2.4296 | 3.0910 | 1.3817 |
| | 1 | 0.0887 | | 0.4888 | | | 0.7070 | 3.8960 | | |
| | 1 | 0.0969 | | 0.5340 | | | 0.2827 | 1.5579 | | |
| | 2 | 0.1410 | | 0.7205 | | | 1.0496 | 5.3633 | | |
| | 2 | 0.1075 | | 0.5493 | | | 0.6217 | 3.1768 | | |
| | 2 | 0.1559 | | 0.7966 | | | 0.4153 | 2.1221 | | |
| R429H | 1 | 0.0440 | | 0.2425 | 0.6518 | 0.3421 | 0.5257 | 2.8970 | 3.3123 | 0.2077 |
| | 1 | 0.1392 | | 0.7671 | | | 0.6221 | 3.4282 | | |
| | 1 | 0.1881 | | 1.0366 | | | 0.6093 | 3.3576 | | |
| | 2 | 0.2011 | | 1.0276 | | | 0.6523 | 3.3332 | | |
| | 2 | 0.0928 | | 0.4742 | | | 0.6704 | 3.4257 | | |
| | 2 | 0.0710 | | 0.3628 | | | 0.6717 | 3.4323 | | |
| L505F | 1 | 0.1397 | | 0.7698 | 0.9190 | 0.3033 | 1.4806 | 8.1591 | 10.2073 | 4.8106 |
| | 1 | 0.0986 | | 0.5434 | | | 1.5169 | 8.3591 | | |
| | 1 | 0.1792 | | 0.9875 | | | 1.1621 | 6.4039 | | |
| | 2 | 0.2713 | | 1.3863 | | | 1.6742 | 8.5549 | | |
| | 2 | 0.1413 | | 0.7220 | | | 1.9623 | 10.0271 | | |
| | 2 | 0.2163 | | 1.1053 | | | 3.8631 | 19.7399 | | |

p values between the wild-type vs empty vector, N459S, R429H, and L505F were $1.3\times10^{-9}$, $2.1\times10^{-8}$, $2.9\times10^{-8}$, $1.3\times10^{-3}$, respectively.

**Supplementary Table 8** SRE Luciferase Reporter Assays

| Plasmid | Experiment | Presence of sterol | | | | | Absence of sterol | | | |
|---|---|---|---|---|---|---|---|---|---|---|
| | | Luciferase | Average of wild-type luciferase | Normalized by A-WT | Average | Corrected SD | Luciferase | Normalized by A-WT | Average | Corrected SD |
| Vector | 1 | 1.6030 | | 0.9786 | 1.0000 | 0.0280 | 1.7060 | 1.0415 | 1.0276 | 0.0249 |
| | 1 | 1.6170 | 1.6380 | 0.9872 | | | 1.6990 | 1.0372 | | |
| | 1 | 1.6940 | | 1.0342 | | | 1.6990 | 1.0372 | | |
| | 2 | 1.7250 | | 0.9942 | | | 1.7950 | 1.0346 | | |
| | 2 | 1.6840 | 1.7350 | 0.9706 | | | 1.6950 | 0.9769 | | |
| | 2 | 1.7960 | | 1.0352 | | | 1.8010 | 1.0380 | | |
| Wild type | 1 | 3.9320 | | 0.9844 | 1.0000 | 0.0363 | 13.9050 | 3.4812 | 3.2080 | 0.2561 |
| | 1 | 3.8300 | 3.9943 | 0.9589 | | | 12.9190 | 3.2343 | | |
| | 1 | 4.2210 | | 1.0567 | | | 13.8270 | 3.4617 | | |
| | 2 | 3.5270 | | 0.9991 | | | 10.4800 | 2.9686 | | |
| | 2 | 3.6270 | 3.5303 | 1.0274 | | | 10.0580 | 2.8490 | | |
| | 2 | 3.4370 | | 0.9736 | | | 11.4850 | 3.2532 | | |
| N459S | 1 | 2.1480 | | 0.9968 | 1.0000 | 0.0242 | 3.7400 | 1.7355 | 1.6103 | 0.1389 |
| | 1 | 2.1170 | 2.1550 | 0.9824 | | | 3.5190 | 1.6329 | | |
| | 1 | 2.2000 | | 1.0209 | | | 3.7410 | 1.7360 | | |
| | 2 | 3.0080 | | 1.0353 | | | 4.7740 | 1.6432 | | |
| | 2 | 2.8190 | 2.9053 | 0.9703 | | | 3.9700 | 1.3665 | | |
| | 2 | 2.8890 | | 0.9944 | | | 4.4960 | 1.5475 | | |
| R429H | 1 | 2.1450 | | 1.0052 | 1.0000 | 0.0458 | 2.5540 | 1.1968 | 1.2467 | 0.0537 |
| | 1 | 2.0120 | 2.1340 | 0.9428 | | | 2.5050 | 1.1739 | | |
| | 1 | 2.2450 | | 1.0520 | | | 2.7650 | 1.2957 | | |
| | 2 | 2.5740 | | 1.0308 | | | 3.076 | 1.2319 | | |
| | 2 | 2.3610 | 2.4970 | 0.9455 | | | 3.208 | 1.2847 | | |
| | 2 | 2.5560 | | 1.0236 | | | 3.239 | 1.2972 | | |
| H171F | 1 | 2.0100 | | 1.0140 | 1.0000 | 0.0321 | 2.1060 | 1.0624 | 1.0693 | 0.0346 |
| | 1 | 1.9030 | 1.9823 | 0.9600 | | | 2.0440 | 1.0311 | | |
| | 1 | 2.0340 | | 1.0261 | | | 2.0670 | 1.0427 | | |
| | 2 | 2.0630 | | 0.9800 | | | 2.3400 | 1.1116 | | |
| | 2 | 2.0580 | 2.1050 | 0.9777 | | | 2.2230 | 1.0561 | | |
| | 2 | 2.1940 | | 1.0423 | | | 2.3400 | 1.1116 | | |

p values between the wild-type vs empty vector, N459S, R429H, and H171F were $3.2 \times 10^{-8}$, $5.1 \times 10^{-7}$, $1.2 \times 10^{-7}$, $5.0 \times 10^{-8}$, respectively.

## Supplementary References


1. Bateman, J.F. & Golub, S.B. Deposition and selective degradation of structurally-abnormal type I collagen in a collagen matrix produced by osteogenesis imperfecta fibroblasts in vitro. *Matrix Biol* **14**, 251-62 (1994).
2. Aten, E. *et al.* Keratosis Follicularis Spinulosa Decalvans is caused by mutations in MBTPS2. *Hum Mutat* **31**, 1125-33 (2010).
3. Araujo, C., Goncalves-Rocha, M., Resende, C., Vieira, A.P. & Brito, C. A Case of IFAP Syndrome with Severe Atopic Dermatitis. *Case Rep Med* **2015**, 450937 (2015).
4. Fong, K. *et al.* Ichthyosis follicularis, atrichia, and photophobia syndrome associated with a new mutation in MBTPS2. *Clin Exp Dermatol* **40**, 529-32 (2015).
5. Izumi, K., Wilkens, A., Treat, J.R., Pride, H.B. & Krantz, I.D. Novel MBTPS2 missense mutation in the N-terminus transmembrane domain in a patient with ichthyosis follicularis, alopecia, and photophobia syndrome. *Pediatr Dermatol* **30**, e263-4 (2013).
6. Haghighi, A. *et al.* A missense mutation in the MBTPS2 gene underlies the X-linked form of Olmsted syndrome. *J Invest Dermatol* **133**, 571-3 (2013).
7. Bornholdt, D. *et al.* Genotype-Phenotype Correlations Emerging from the Identification of Missense Mutations in MBTPS2. *Hum Mutat*, 10.1002/humu.22275 (2013).
8. Kraenzlin, M.E., Kraenzlin, C.A., Meier, C., Giunta, C. & Steinmann, B. Automated HPLC assay for urinary collagen cross-links: effect of age, menopause, and metabolic bone diseases. *Clin Chem* **54**, 1546-53 (2008).
